# Desiccation Tolerance of Epiphytic Macrolichens in an Evergreen Temperate Rain Forest (Alerce Costero National Park, Chile)

**DOI:** 10.3390/plants13111519

**Published:** 2024-05-31

**Authors:** Johana Villagra, José Raggio, David Alors, Leopoldo G. Sancho

**Affiliations:** 1 Departamento de Ciencias Agropecuarias y Acuícolas, Facultad de Recursos Naturales, Campus San Juan, Pablo II, Universidad Católica de Temuco, Temuco 478 0694, Chile; 2 Departamento de Biología y Químicas, Facultad de Recursos Naturales, Campus San Juan Pablo II, Universidad Católica de Temuco, Temuco 478 0694, Chile; 3 Departamento de Farmacología, Farmacognosia y Botánica, Facultad de Farmacia, Universidad Complutense de Madrid (UCM), 28040 Madrid, Spain; jraggioq@ucm.es (J.R.); sancholg@farm.ucm.es (L.G.S.)

**Keywords:** epiphytic macrolichens, desiccation tolerance, chlorophyll fluorescence, Alerce Costero National Park

## Abstract

The Valdivian region has a temperate rainy climate with differences in rainfall throughout the year. This heterogeneity results in periods of summer drought that expose the poikilohydric epiphytes to desiccation. With this research, we aim to answer different research questions related to phorophyte preference, response to desiccation, and response to radiation. How does the diversity of macrolichens vary at a local and microclimate scale in three tree species within an evergreen forest? What is the tolerance limit of macrolichens against prolonged desiccation, according to evaluation of the maximum efficiency of PSII (Fv/Fm) and pigment concentration? What is the tolerance limit against a potential increase in radiation? We found that macrolichen communities are determined by tree species, which regulate the suitability of the substrate by modifying the temperature and humidity conditions. In addition, our results show a rapid photosynthetic alteration in temporal exposure to desiccation, measured through Fv/Fm and pigment concentration. Our results showed that the most sensitive lichens to radiation and desiccation are not coincident. We confirm the low tolerance of macrolichen species to high radiation, reflected in the saturation profile obtained for the set studied. The lichen community in the evergreen forest showed high complexity and vulnerability, pointing to the importance of more research.

## 1. Introduction

The evergreen forests of the Cordillera de la Costa in Chile are a very humid and shady ecosystem that is threatened by continuous anthropogenic intervention including the replacement of native species with agricultural and forestry plantations [1]. The importance of the Chilean evergreen forest lies in its unique floristic composition [2,3] characterized by a rich diversity and endemism of epiphytic lichens [4]. The lichens in the evergreen forest reach a high biomass that make them a visible and important component of the forest ecosystem [4], which is possible due to an optimal combination of water regime and temperature [5], constituting a relevant contribution to local biodiversity [4,6,7]. Epiphytic lichens are an important group of organisms in forest ecosystems [8,9] and show high specificity to host species and the microhabitats offered by the phorophyte trees [10,11]. Lichens are poikilohydric organisms and therefore lack mechanisms that regulate the absorption and loss of water [12]. They are morphologically [13,14] and physiologically [15] adapted to changes in their water content, therefore being especially sensitive to climatic conditions [16]. For this reason, changes in the local climate can cause modifications in the composition of epiphytic communities in host trees [17] or even the disappearance of sensitive species [18] with a consequent decrease in diversity of species as a result of failure to respond fast enough to habitat changes [19]. Generally, lichens are highly tolerant to desiccation; an excellent example of this survival capacity has been recorded in lichens exposed to outer space conditions [20,21]. Survival times after long periods of desiccation have also been recorded in *Ramalina maciformis*, with more than one year of exposure to desiccation at 1% water content [22,23]. However, species adapted to shady forest habitats with high relative humidity have been shown to be highly sensitive to desiccation [24]. An example is the shade-adapted lichen *Pseudocyphellaria dissimilis* from the New Zealand rain forest, which shows high sensitivity when exposed to short periods of desiccation. Kranner et al. [25] evaluated tolerance of desiccation through determining the chlorophyll loss in three lichens after a period of 9 weeks dehydration. The authors found that in the sensitive species *Lobaria pulmonaria* and *Peltigera polydactyla*, net photosynthesis needed a long time to recover in correlation with chlorophyll loss, while the tolerant species *Pseudevernia furfuracea* did not show chlorophyll decrease and photosynthesis recovered after a short time period. In addition to desiccation, lichens may be particularly sensitive to high light stress as well [26]. Kershaw and MacFarlane [27] reported that populations of *Peltigera aphthosa* collected from the dense shade of spruce in northern Ontario were extremely sensitive to high light, while in contrast, populations collected from open habitats were much more tolerant. Especially, shade-adapted forest lichens showed lower resistance to high radiation with a strong decrease in photosynthetic efficiency (Fv/Fm) when light intensity increased [28,29,30]. In this sense, epiphytic lichens from shaded forests are important environmental indicators because of their sensitivity to land-use and habitat changes [31], decreasing species richness, and the increasing number of species threatened mainly by habitat degradation caused by human interference [32].

The inappropriate management of ecosystems can alter the biodiversity and balance of epiphytic lichens, with the final result of diversity loss [28]. For this reason, it is important to understand the response of these epiphytes to environmental fluctuations and to predict their response to the adversity predicted by global climate change. If tolerance limits and adaptive capacity are exceeded, stress can cause permanent damage and even the death of these epiphytes [33]. This raises the question: how much desiccation can these epiphytes tolerate in response to environmental conditions in a context of climate change or habitat management that generates impacts on water availability? Productivity in lichens can vary from zero to a maximum of activity in a few minutes depending on the available water [34]. In the context of climate change, minimum and maximum temperatures are projected to increase in the entire Chilean territory throughout all seasons, with a winter precipitation decrease exceeding 40% by the end of the twenty-first century [35]. These projections represent a threat to water resources and natural vegetation, with negative consequences especially for species that need greater availability of water in the environment to maintain their active metabolism. Functional studies that evaluate desiccation and the photosynthetic activity of lichens in Chile are few [36], even faced with the constant threat of climate change in the country [37]. The objective of this work is to characterize the abundance of macrolichens and their preference for host trees within the same evergreen vegetation unit. We also aim to study the response of photosynthetic capacity associated with the decrease in water availability and the response to the light effect in species with different morphology within the same forest unit. The results obtained in this work will help in the understanding of the responses of these important lichen communities to predicted climate change and to different degrees of forest management.

## 2. Results

### 2.1. Diversity and Distribution of Macrolichens

We found a total of six families, eight genera, and 13 species of macrolichens (Table 1); the highest diversity was recorded in the Lobariaceae family with 7 species belonging to two genera. The highest species richness was recorded on *Nothofagus nitida* (Nn), differing statistically from *Saxegothaea conspicua* (Sc) and *Drimys winteri* (Dw) (Kruskal–Wallis ANOVA on ranks *p* < 0.001; H = 30.895; d.f. = 2). *N. nitida* had the highest diversity (0.89) (Shannon–Wiener index), followed by the *S. conspicua* (0.57) and *D. winteri* (0.43). Regarding the coverage percentage, no significant differences were recorded between the different tree species (*p* = 0.21, ANOVA, α = 0.05). The Sorensen’s index resulted in a 67% similarity between *N. nitida* and *S. conspicua*, sharing 7 species of a total of 13. In *N. nitida* and *D. winteri*, the Sorensen’s index showed a 44% similarity.

When the lichen community was analyzed along the height of the tree trunk, significant differences in species richness were found only between the *Drimys winteri* strata (Kruskal–Wallis ANOVA on ranks; *p *< 0.001; H = 46.7; d.f. = 5). No significant differences were found in terms of relative coverage by strata in the other two phorophytes (Kruskal–Wallis ANOVA on ranks; *p *= 0.29; H = 7.13; d.f. = 5), however, a high coverage of *Sticta ainoae* was recorded on stratum A of *N. nitida* (Nn A 63.52 ± 4.87, Table 2).

### 2.2. Microclimate Measurements

Mean relative humidity was higher in *N. nitida* (*p* = 0.0014) than *S. conspicua* and *D. winteri* (89.7± 0.28, 88.6 ± 0.33, 88.3 ± 0.3 respectively). We did not find differences in temperature values between tree species (*p* = 0.48; *N. nitida* 10.1 ± 0.12, *S. conspicua* 9.82 ± 0.12, and *D. winteri* 9.83 ± 0.12). Vapor pressure deficit (VPD) presented no significant differences between the tree species (*p* = 0.87). Table 3 shows the microclimatic values per stratum; relative humidity in the three tree species was higher in stratum A (0–0.5 m from the ground) than in stratum B (0.5–1.8 m from the ground). The temperature values were statistically higher in stratum B in *N. nitida* and *D. winteri*. Finally, VDP increased with height but was only significant in *N. nitida* and *S. conspicua* (*p* > 0.001 in both trees).

### 2.3. Desiccation Tolerance

**Figure 1:**
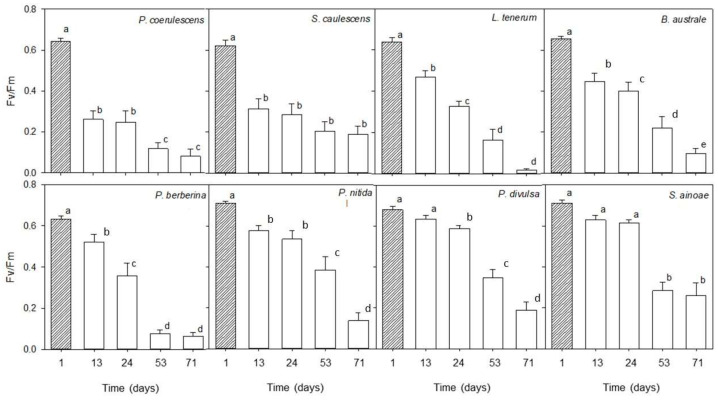
Maximum quantum yield of Photosystem II (Fv/Fm) in 8 epiphytic macrolichens during thallus dehydration. ANOVA with repeated measures was used to compare Fv/Fm over time, ANOVA *p *≤ 0.05, followed by Tukey’s HSD post hoc test. Mean ± SE (*n* = 12). Different letters (a–e) show statistical differences.

Figure 1 summarizes the temporal evolution of potential yield of photosynthetic processes in Photosystem II (Fv/Fm, from day 1 to day 71 of the treatment). Fv/Fm values at time 1 were greater than 0.6 in chlorolichens and cyanolichens; therefore, we consider that the photosynthetic apparatus of the eight evaluated species was in optimal condition before collecting them. Also, it was shown that from day 13, there was a significant depression in Fv/Fm in most species with respect to the physiological state of the starting point (*p* < 0.001, ANOVA, α = 0.050). We found that the cyanolichens *Pseudocyphellaria coerulescens* and *Sticta caulescens* showed the greatest percentage of Fv/Fm decline at 13 days after collection from the field (57.5%, 49.1% respectively), followed by fruticose chlorolichens *Bunodophoron australe* (31.6%) and *Leifidium tenerum* (26.2%) and foliose chlorolichens *Pseudocyphellaria nitida* (18.6%) and *Pseudocyphellaria berberina* (17.7%). Otherwise, *P. divulsa* and *S. ainoae* were less affected during the first weeks of treatment. In both species, Fv/Fm declined from day 53 (6.3 and 11.4% respectively).

Concentration of photosynthetic pigments over time was different between the species (Supplementary Table S1). At time 1, the species with the highest concentrations of chlorophyll were *Sticta ainoae* (2.22 mg/g) and *Pseudocyphellaria berberina* (2.15 mg/g) (Kruskal–Wallis ANOVA on ranks; *p* = 0.003; H = 21.653; d.f. = 7). With respect to carotene concentration, the species with the highest concentration at time 1 was *P. berberina* (2.47 mg/g), followed by *S. ainoae* (2.02 mg/g) and *P. divulsa* (1.35 mg/g). The carotene content of the two Pseudocyphellaria species was statistically different but not different from the carotene content of *S. ainoae*, and *P. divulsa* carotene content was not different from the five other species analyzed (Kruskal–Wallis ANOVA on ranks; *p* = 0.002; H = 22.897; d.f. = 7). The pigment content (chlorophyll and carotene) over time during the desication treatment showed three patterns within species: (1) decrease after 53 days in *L. tenerum* and *P. berberina*; (2) decrease after 71 days in *B. australe*, *P. coerulescencs*, and *S. ainoae*; (3) and the maintaining of pigment content over 71 days in *Sticta caulescens*, *P. divulsa*, and *P. nitida*.

### 2.4. Response to Light

We measured Y(II) as an indicator of the efficiency of reaction centers under lighting conditions of three relevant radiations (24, 55, and 367 μmol m^−^^2^ s^−^^1^) to unravel how the set of lichens were adapted to different light intensities. From an initial value of Y (II) greater than 0.6, all the lichen species tested decreased at 24 PPFD, showing a percentage of Y(II) with respect to the initial value which was not different between species (alpha 0.05). At 55 PPFD, the percentage of decrease in Y(II) varied significantly between species (*p *= 0.026). For example, *P. divulsa* showed the greatest decay (78.1%), being statistically different from *S. caulescens *and *P. nitida*, which decreased by 49.02% and 53.81%. The rest of the species showed Y (II) decays at 55 PPFD that were not statistically different from each of the other species. At 367 PPFD, the yield percentages did not show significant differences between species (*p *= 0.070).

In situ ETRmax values (Supplementary Table S2) differed between the different species (*p*= 0.016, ANOVA, α = 0.050). The maximum ETR values were recorded for *P. coerulescens*, *S. caulescens*, and *P. divulsa* (122 μmol m^−^^2^ s^−^^1^). The rest of the evaluated species presented maximum ETR values at 183 μmol m^−^^2^ s^−^^1^. In situ PPFDsat values differed between the different species (*p *= 0.023, ANOVA, α = 0.050). The range of the saturation point for the macrolichens of the mixed forest fluctuated between 24.73 ± 8.51 and 87.09 ± 23.2 μmol m^−^^2^ s^−^^1^ (*L. tenerum* and *P. nitida*, respectively).

## 3. Discussion

The relationship between tree species and the diversity of lichen species is a known phenomenon [8]. The importance of these epiphytes in the Chilean evergreen forest lies in their rich diversity, high biomass, and endemism [4], which is possible due to the area’s water regime, among other factors [5]. However, these conditions could also be unfavorable for some lichens, since they would be limited in terms of the presence of species by other groups, such as bryophytes, which dominate better in these conditions [38]. Our results coincide with this affirmation, since the number of species recorded in this study (14 species) was lower than those recorded in areas of greater exposure such as *Sphagnum* and pulvinated peat bogs (35 and 23 species, respectively [39]), *Nothofagus pumilio* (19 species [40]), and *Araucaria araucana* (more than 30 species [41]). However, the diversity of the lichens that were found is represented by 57% endemism and sharing 20% of the genera on the trunk and branches stratum described by Galloway [4] for the temperate rain forest of southern Chile.

We found that the structure of the macrolichen community was determined by the tree species, since this regulates the suitability of substrate by modifying the temperature, humidity, and light conditions on which the epiphytes depend. Thus, species richness of epiphytic macrolichens varies significantly between host tree species. The largest number of exclusive species was found on *N. nitida *(especially cyanolichens, for example, *Collema leave*, *Sticta caulescens*, *Sticta hypocra*). *D. winteri* presented two exclusive species within the unit (*Lepolichen coccophorus* and *Platismatia glauca*) which have been related to a greater range of substrates, mainly in environments with greater light intensity [42,43,44]. On the other hand, although we found significant differences in terms of only the vertical distribution of the species in *D. winteri*, our data indicate preferences of certain species regarding the microenvironments offered by the phorophyte. Thus, we found that the largest number of total species and chlorolichens occurred in the B stratum of the three tree species. This coincides with different studies that show a pattern through the vertical gradient, in which the number of epiphytes tends to increase from the base of the tree to the canopy [11,45,46]. Such a pattern would respond to fluctuating microclimatic conditions throughout the tree [47,48,49] and probably reflects a set of ecophysiological requirements necessary to adapt to the environment [50]. On the other hand, the greatest number of cyanolichens were found in the darkest and most humid areas (A stratum), an association that could turn out to be highly fragile, since shade epiphytes are more sensitive to environmental changes, mainly because they have lower tolerance to photoinhibition when exposed to more sunlight [51,52,53,54]. In addition, cyanolichens would be favored by the formation of small pools of water observed at the base of tree species as a result of stem runoff. This would favor cyanolichens that require liquid water to start the photosynthetic process [34]. The cyanolichen species *Pseudocyphellaria coerulescens* and *Cora glabrata* have been recorded in areas with more exposure to light in temperate forests of *Nothofagus nitida* [55], which showed the highest humidity within the three tree species (Table 3). We also found a group of generalist species including *Pseudocyphellaria nitida* and *Pseudocyphellaria berberina* that were able to occur in all three tree species and both strata, with the species *L. tenerum* occurring on the B stratum of all three tree species. (Table 2). These species, despite inhabiting an area of high humidity [5], probably have moderate water requirements in comparison with species restricted to the lower stratum of *N. nitida* and *Saxegothaea conspicua*. Another group of species associated with more exposed areas of the forest occurred in the B stratum of *D. winteri*. The dominant species of this group was *P. berberina, *accompanied by *P. glauca* and *L. coccophorus *(which were recorded only in the B stratum of *D. winteri*) among other lichen species (Table 2). This set of epiphytes has been related to a wide range of substrates, but mainly to higher light intensity [5]. Thus, lichen colonization responds to a series of conditions determined by the heterogeneity of the microclimate [47,49,56,57].

All the lichens showed a decay of maximum quantum yield in Photosystem II (Fv/Fm) as a consequence of the dehydration stress (Table 4). We observed species-specific responses in terms of Fv/Fm decrease, which can be explained in terms of functional groups (cyanolichens, fruticose chlorolichens, and foliose chlorolichens). The most sensitive species to dehydration were the cyanolichens *Pseudocyphellaria coerulescens* and *Sticta caulescens*, which were minority components of the B strata of *S. conspicua* and *Drimys winteri* and the A strata of *S. conspicua* and *N. *nitida, respectively. *Pseudocyphellaria coerulescens* and *Sticta caulescens* showed decreases in Fv/Fm of 59% and 50%, respectively, after thirteen days. This was in accordance with the observed higher sensitivity to environmental stress among cyanolichens [25,58] that, in contrast to the green algal species, do not show net photosynthesis when hydrated by water vapor alone but require liquid water [16,59,60,61]. The fruticose lichens *Bunodophoron australe* and *Leifidium tenerum*, which were found only in the B strata, showed higher sensitivity to dehydration (31.7% and 26.5% FvFm decay, respectively) compared with foliose lichens. This is in accordance with previous works that have shown that the fruticose biotypes are highly sensitive to disturbance to habitat fragmentation via induced edge effects, dispersal of pollutants by pesticide drift, and air pollution sensitivity [62,63]. Foliose biotypes were found in both A and B strata and were found to be more tolerant to dehydration measured as Fv/Fm decay, with *Pseudocyphellaria nitida* showing a decay of 18.7% and *Pseudocyphellaria berberina* 17.5% after 13 days. Even more dessication tolerance was shown by the other two foliose chlorolichens, *Pseudocyphellaria divulsa* and *Sticta ainoae*, which showed no significant decay in this parameter until day 53. Cyanolichens were shown to be the most sensitive to dehydration measured as Fv/Fm decay, supporting the use of this group of lichens as indicators of good quality of the environment and the presence of liquid water [64]. Our results support the hypothesis that epiphytic lichens of the Valdivian rain forest are adapted to short periods of desiccation, but do not survive if desiccation is prolonged over time.

The photosynthetic pigment contents measured in this work were within the range measured in chlorolichens of New Zealand´s temperate rainforest [65]. It should be mentioned that the pattern of decay observed in chlorophyll was coupled to the decay observed in carotene. Our results showed significant decreases in photosynthetic pigments at different days of desiccation within species: in *P. berberina* after 24 days, in *L. tenerum *and *P. berberina* after 53 days, and *B. australe*, *P. coerulescencs*, and *S. ainoae* needed 71 days of desiccation, while the species *Sticta caulescens*, *P. divulsa*, and *P. nitida* did not show differences in pigment concentration over time. Our results showed generalized and faster Fv/Fm reduction with desiccation over time, with six species showing significant decay after 13 days, the decay after 24 days in *P. divulsa*, and after 53 days in * S. ainoae*. Therefore, Fv/Fm preceded the reduction in the content of photosynthetic pigments, and the degradation and damage to photosynthetic pigments should be considered not only in a quantitative way but also in a qualitative way.

The studied lichens from Valdivian Forest showed great performances with 24 μmol m^−2^ s^−1^ and a decay in 50% of the photosynthetic yield when a light of 55 μmol m^−2^ s^−1^ was applied. These results suggest an adaptation to low radiation in the case of evergreen forest lichens, coinciding with the results of Green et al. [65], who found lower light saturation points in lichens from shaded habitats in New Zealand compared with those from sun-exposed habitats.

The saturation point is an important parameter showing the light intensity, which allows positive photosynthetic balance, and the values obtained in this study are in concordance with those obtained from lichens adapted to shade conditions inside forests, as reported by Lakatos et al. [14] and Pardow et al. [66], contrasting with the high tolerance to light (higher saturation points) shown in lichens inhabiting exposed areas [67,68]. The measured decrease of photosynthetic yield from low light intensities and the low saturation points reflect adaptation to their shady environment. It has been shown that for lichens inhabiting shady forests, the low indirect light could be enough to stimulate lichen growth [69], as could the direct light experienced for short periods as sunflecks [14]. On the other hand, exposure to higher light intensities negatively affects the growth of lichens from shady rain forests [70]. Thus, although light is essential for the photosynthesis of these organisms, an excess of energy absorbed by photobionts can cause irreversible photodamage in the algal Photosystem (PS) II [71].

The saturation point measured for the species studied reached the highest value for *P. nitida* (87.09) and was statistically different from the three species *L. tenerum, P divulsa*, and *P. coerulescens*. *P. nitida* was also the species which showed the highest ETR value; the relationship between ETR and incident PAR indicates the level at which the photosynthetic apparatus suffers due to excessive radiation, as the higher the ETR value is, the higher is the light intensity necessary to affect negatively the photosynthetic apparatus. As *P. nitida* showed the highest saturation point and the highest ETR value, this species is considered the least sensitive to light among the species measured. This result is in accordance with its high success measured as coverage within the three tree species and the two strata, greater than 25% of the average cover (Table 2).

It is remarkable that in all species, the maximum ETRmax values were not obtained at maximum radiation, which suggests generalized photoinhibition. The ETR max values were in the ranges reported by Pardow et al. [66] for crustose lichens from tropical forests (between 4.6 and 1.8) and Lakatos et al. [14] for crustose lichens in a evergreen forest in French Guiana (between 7.52. and 14.8). In contrast, the highest ETRmax values have been reported in light-exposed areas for the species *Stereocaulon foliolosum* (45) and *Lecanora muralis* (179) [59].

We showed an important decay of photosynthetic potential in response to dehydration and low light saturation points in most evergreen forest lichens. To a certain extent, these findings allow us to predict the vulnerability of species with high water requirements or low radiation tolerance in the face of a scenario of water stress expected in the region in the context of climatic oscillations and global warming [60,61]. It is worth highlighting the importance of developing long-term eco–physiological studies that allow us to understand the response and adaptation of these organisms that are especially vulnerable to the effects of climate change.

## 4. Materials and Methods

### 4.1. Study Area

**Figure 2:**
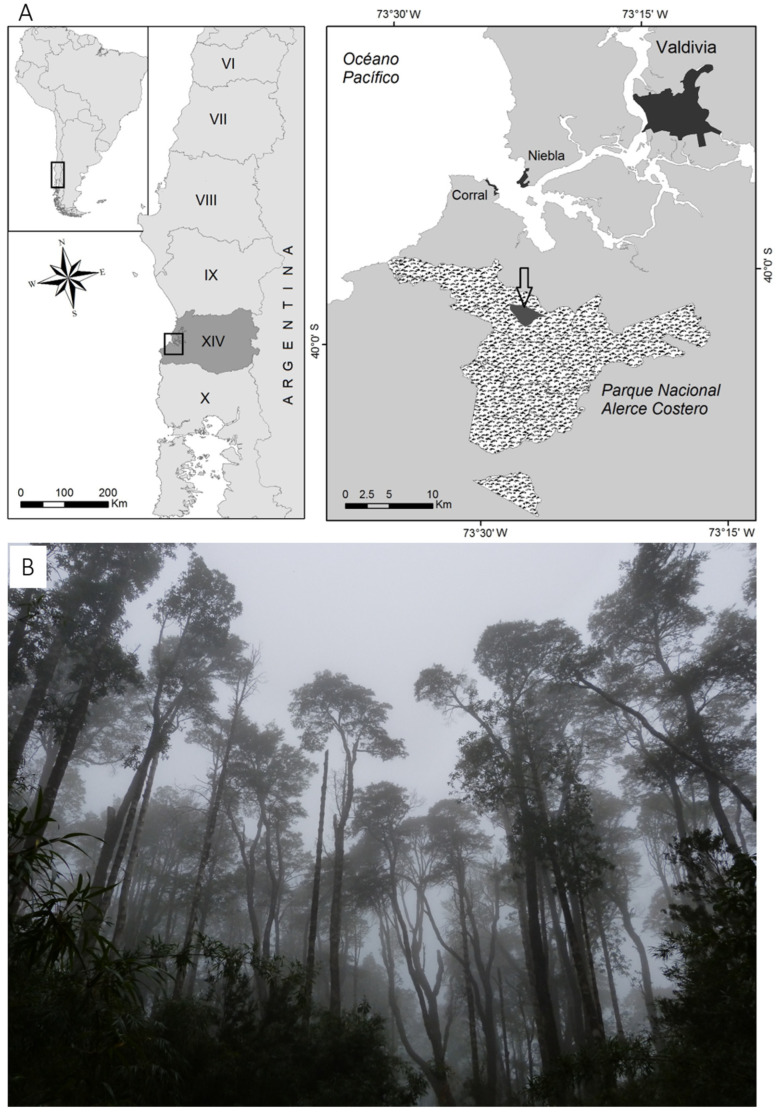
(**A**) Location of the study area in the Alerce Costero National Park (Los Ríos Region, XIV Region, Chile). (**B**) Evergreen Forest of *Nothofagus nitida*, *Saxegothaea conspicua*, and *Drimys winteri* (mixed forest in the text).

The specimens were collected in an evergreen forest (hereinafter called mixed forest) Alerce Costero National Park (40°00′ S/73°25′ W, 600 m altitude), Los Ríos Region, Chile (Figure 2A,B). The climate of the area is defined as hyper-humid mesotemperate [62]. The mean annual temperature is 10 °C, the warmest month is January with 17.2 °C, and the coldest July, with 7.6 °C [63]. The region has a mean annual rainfall of 2130 mm, registering maximums of 4000 and 5000 mm per year [72,73] and, exceptionally, rainfall of around 7000 mm in the year 2000 [5].

### 4.2. Censuses of the Lichen Flora

We selected five 30 × 30 m plots, each 50 m apart, to study the macrolichens in the stands of *Nothofagus nitida*, *Saxegothaea conspicua*, and *Drimys winteri* species dominant in the evergreen forest selected. Within each plot, we selected five old trees per phorophyte species, for a total of 45 trees. The censuses were carried out through applying a 20 × 50 cm quadrant directly to the trunk to evaluate the cover percentage. Two censuses were made, with random orientation from ground level to 0.5 m (stratum A) and from 0.5 to 1.80 m (stratum B). The identification of the species was conducted based on [55,74,75,76]. For the richness, coverage, vertical distribution, and community diversity analysis of the lichen, the program PAST 4.11 was used. For each subsample studied, species richness was registered and the Shannon–Wiener diversity index (H′) was calculated according to the formula:

$$H^{\prime }=-\sum_{i=1}^{n}=piln(pi)$$

where pi = relative proportion (coverage) of the i species.

### 4.3. Microclimate

Six temperature/humidity loggers (iButton^®^ DS1923, MCI electronics, Santiago, Chile) were installed in the study area, programmed to record every 4 h. The recorded period was from 5 September 2014 to 18 February 2015. In this study, 2 sensors were placed on 1 specimen of each tree species (the loggers were placed uniformly on A and B strata). From the data obtained on humidity and temperature, the water deficit was estimated. Saturation pressure vapor in the air (SPV) was calculated as in Murray (1967) using the equation SPV Pascals = 610.7 × 107.5T/(237.3 + T), and vapor pressure deficit (VPD) was calculated from T and RH according to the following equation: VPD (Pascals) = (1 − (RH/100) × SPV), where VDP is an approximate measure of the drying power of the air.

### 4.4. Sample Collection

A total of 20 individual lichen samples were collected of each of the following species: *Pseudocyphellaria coerulescens*, *Leifidium tenerum*, *Bunodophoron australe*, *Pseudocyphellaria berberina*, *Pseudocyphellaria nitida*, *Pseudocyphellaria divulsa*, *Sticta ainoae *and *Sticta caulescens*. These species were selected according to coverage percentage within the study area (Table 1). Samples were dried in a room with a natural air flow for 2 days, stored for 5 days in a paper envelope, and transported to the laboratory.

### 4.5. Desiccation Tolerance

The initial measure of photosynthetic response started in situ at time 1 and was considered as the control. The following measurements were taken after maintaining the lichens in a climatic chamber simulating desiccation conditions. The programed conditions simulated day/night: 15 °C and 60% humidity for 12 h with photosynthetically active light (PPFD) at 100 μmol m^−2^ s^−1^ and 12 °C and 55% humidity for 12 h in darkness (FITOTRON^®^ SGC 120, High Specification Plant Growth Chambers, Weiss Technik UK, Loughborough, UK). We measured two parameters to evaluate desiccation tolerance: maximum quantum yield of Photosystem II (Fv/Fm), was used as an indicator of potential photosynthetic performance of the samples after preadaptation to darkness, along with pigment content (total chlorophyll and carotenes). First, for the evaluation of Fv/Fm, four individuals (4 replicates) were considered and three measurements (3 pseudo replicates) per individual were taken (*n *= 12). From the same individuals, the pigment content was evaluated (*n *= 4). After 13, 24, 53, and 71 days, four specimens of each of the 8 species were removed from the chamber and hydrated to saturation with mineral water for 30 min. Samples were moistened, left in the dark for 20 min, then illuminated for 1 s with a saturating excitation pulse (1800 μmol s^−1^ m^−2^), and Fv/Fm was measured using a fluorometer MINI-PAM (Walz, Effeltrich, Germany). The fluorometer was positioned at 2–4 mm with an angle of 60° to avoid shading the thallus [77].

The chlorophyll content of the 8 lichen species was determined at the same sampling times as Fv/Fm, following [78]. A total of 20 mg for each sample was repeatedly washed with CaCO_3_-saturated acetone to remove lichen substances, and pigments were extracted in CaCO_3_-saturated DMSO. Absorbance of the extracts was measured using a UV–visible spectrophotometer (Uvikon XL, NorthStar Scientific, Leeds, UK). Absorbance was measured at 664.9, 648.2, 480, 435, and 415 nm, and total chlorophyll concentrations calculated (expressed in mg g^−1^ dry weight) using the formulas provided in Barnes et al. [78].

### 4.6. Response to Light

A rapid light curve (RLC) was measured to obtain the evolution of the quantum use efficiency of PSII in the light (YII or yield) versus increasing levels of radiation and to evaluate the level of light at which Photosystem II was saturated (PFDsat) [77,79]. RLC per sample was performed in situ and 8 light intensities were applied to each RLC, which increased every 10 s (24, 38, 55, 81, 122, 183, 262, and 367 µmol m^−2^ s^−1^). The effective quantum efficiency of Photosystem II (Y II) and the ETR were measured after acclimation in darkness for 20 min. Each Φ PSII measurement was used to calculate the electron transport rate (ETR) through Photosystem II using the following equation: ETR = PPFD × 0.84 × 0.5 × Y(II), where PPFD is the photosynthetic PPFD recorded with the sensor in the leaf clip, 0.84 is the estimated mean proportion of incident light absorbed by the photosystems, 0.5 is the required factor for both photosystems to account for absorbed photons, and Y(II) is the effective quantum yield of Photosystem II [80]. The values for Y(II) oscillated between 0.76 and 0.6 when environmental stress was negligible for chlorolichens and between 0.61 and 0.41 in cyanolichens, in agreement with Jensen et al. [81], but these values decreased with increasing environmental stress. For each specimen, the data for Y(II) and ETR against PFD were adjusted according to the statistical models proposed by Rascher et al. [77]. Light saturation points were determined with the adjusted ETR vs. PFD curve (calculated as the PPFD value when 90% of the maximum ETR was reached).

### 4.7. Statistical Analysis

For measurements of Fv/Fm, repeated measures, ANOVA was performed. With respect to chlorophyll and carotene content, Y (II), and ETRmax, ANOVA was performed followed by Tukey HSD post hoc tests, indicating significant differences at *p *< 0.05. Analyses were carried out using Statsgraphic (5.1). Data were transformed, if required, to comply with the assumption of normal distribution [82].

## Figures and Tables

**Table 1:** List of macrolichens and percentage of relative cover (CR) registered on *Nothofagus nitida* (Nn), *Saxegothaea conspicua* (Sc), and *Drimys winteri* (Dw), Alerce Costero National Park, Chile.

Family	Species Name	Distribution	Nn	Sc	Dw
Coccotremataceae	* Lepolichen coccophorus *	Endemic			2.8
Collemataceae	* Collema laeve *	Austral	1.3		
Lobariaceae	* Pseudocyphellaria berberina *	Endemic	7.2	22.0	48.0
	* Pseudocyphellaria coerulescens *	Endemic		5.3	8.7
	* Pseudocyphellaria divulsa *	Endemic	20.4	27.6	6.9
	* Pseudocyphellaria nitida *	Endemic	17.8	31.5	25.9
	* Sticta ainoae *	Endemic	34.4		
	* Sticta caulescens *	Endemic	3.9	0.84	
	* Sticta hypochra *	Endemic	1.5		
Parmeliaceae	* Platismatia glauca *	Cosmopolitan			5.6
Sphaerophoraceae	* Bunodophoron australe *	Austral	4.6	5.3	
	* Bunodophoron ramuliferum *	Austral	1.5		
	* Leifidium tenerum *	Austral	6.1	7.5	2.2
Atheliaceae	* Cora glabrata *	Cosmopolitan	1.3		

**Table 2:** Percentage of Relative Coverage (%RC) of macrolichen species represented by tree species (*Nothofagus nitida*: Nn, *Saxegothaea conspicua*: Sc, and *Drimys winteri*: Dw), and two strata analyzed (Stratum A: 0–0.5 m from the ground and stratum B: 0.5–1.8 m from the ground).

Macrolichen Species	Tree Species		
	Nn	Sc	Dw
	Stratum A %RC		
*S. ainoae*	63.52 ± 4.87	Not found	Not found
*S. hypocra*	2.87 ± 2.39	Not found	Not found
*C. laeve*	2.87 ± 4.41	Not found	Not found
*S. caulescens*	7.38 ± 0.76	2.04 ± 2.50	Not found
*P. divulsa*	14.75 ± 6.00	44.22 ± 2.90	20.37 ± 2.50
*P. nitida*	8.61 ± 3.15	29.93 ± 5.20	30.56 ± 11.68
*P. berberina*	Not found	23.81 ± 5.67	49.07 ± 4.29
Macrolichen species	Stratum B %RC		
*B. ramuliferum*	2.74 ± 5.00	Not found	Not found
*S. ainoae*	1.37 ± 2.50	Not found	Not found
*C. laeve*	2.74 ± 2.89	Not found	Not found
*D. glabratum*	1.37 ± 0.00	Not found	Not found
*B. australe*	9.59 ± 2.81	8.96 ± 4.00	Not found
*P. divulsa*	26.48 ± 6.05	16.04 ± 5.95	Not found
*P. nitida*	27.85 ± 4.91	32.55 ± 3.26	23.47 ± 3.98
*P. berberina*	15.07 ± 4.59	20.75 ± 3.90	47.42 ± 3.50
*L. tenerum*	12.79 ± 4.63	12.74 ± 6.63	3.29 ± 2.50
*P. coerulescens*	Not found	8.96 ± 4.41	13.15 ± 7.68
*L. coccophorus*	Not found	Not found	4.23 ± 3.75
*P. glauca*	Not found	Not found	8.45 ± 7.64

**Table 3:** Relative humidity (%), temperature (°C), and Vapor pressure deficit (VPD). A stratum (0–0.5 m from the ground); B stratum (0.5–1.8 m from the ground) by tree species (*Nothofagus nitida*: Nn, *Saxegothaea conspicua*: Sc, and *Drimys winteri*: Dw). *n* = 5 includes standard error.

Tree Species	Humidity (%)			Temperature (°C)			Vapor Pressure Deficit (VPD)		
	A	B	*p*	A	B	*p*	A	B	*p*
Nn	91.6 ± 0.2	87.8 ± 0.4	>0.001	9.8 ± 0.1	10.3 ± 0.1	0.0009	19.3 ± 0.007	29.9 ± 0.01	>0.001
Sc	91.6 ± 0.3	85.6 ± 0.4	>0.001	9.7 ± 0.1	9.9 ± 0.1	0.4	19.9 ± 0.009	34 ± 0.01	>0.001
Dw	89.1 ± 0.3	87.5 ± 0.3	0.002	9.7 ± 0.1	10.0 ± 0.1	0.002	25.1 ± 0.01	29.4 ± 0.01	0.16

**Table 4:** Effective photosynthetic yield of Photosystem II or Yield Y(II) and yield percentage with respect to the initial yield value at different Photosynthetic Photon Flux Density (PPFD) of 24, 55, and 367 μmol m^−^^2^ s^−^^1^. Statistical differences were performed using ANOVA *p* ≤ 0.05 between the different species. Mean ± SE (*n *= 4). Different letters show statistical differences within columns.

Species	Y (II)	PPFD 24	PPFD 55	PPFD 367
	*p* ≤ 0.01	*p* = 0.04	*p* = 0.026	*p* = 0.070
*P. coerulescens*	0.64 ± 0.015	84.42 ± 14.7 a	41.06 ± 4.9 ab	4.66 ± 1.3 a
*S. caulescens*	0.62 ± 0.027	78.42 ± 16.8 a	50.98 ± 11.7 b	4.80 ± 1.3 a
*L. tenerun*	0.64 ± 0.02	89.61 ± 19.5 a	39.09 ± 4.9 ab	3.17 ± 0.7 a
*B. australe*	0.66 ± 0.011	76.26 ± 13.9 a	37.26 ± 4.7 ab	4.34 ± 1.4 a
*P. berberina*	0.63 ± 0.014	70.96 ± 9.6 a	29.16 ± 3.6 ab	4.85 ± 0.5 a
*P. nitida*	0.70 ± 0.011	83.50 ± 10.5 a	46.19 ± 3.6 b	7.93 ± 1.5 a
*P. divulsa*	0.68 ± 0.017	71.43 ± 16.2 a	21.86 ± 3.7 a	2.44 ± 0.5 a
*S. ainoae*	0.68 ± 0.017	68.30 ± 12.2 a	22.74 ± 1.6 ab	3.65 ± 0.8 a

## Data Availability

Data are contained within the article.

## Supplementary Materials

The following supporting information can be downloaded at: https://www.mdpi.com/article/10.3390/plants13111519/s1, Table S1: Concentration of total chlorophyll (Chl) and carotenes (car) in epiphytic macrolichens in the time (1, 13, 24, 53, 71 days of experiment). The pigment concentration is referred as mg/g. Mean ± SE (*n* = 4). ANOVA with repeated measures was used to compare concentration pigment over time and different letters indicate significant differences. ANOVA p≤ 0.05, followed by Tukey’s HSD post-hoc test. Mean ± SE (*n* = 12); Table S2: Maximum electron transport rate (ETRmax) and saturation points (PPDsat) obtained from the exponential fit (using the Sigma Plot Program) of the data recorded in situ. The PPFD units are referred to as µmol m^−2^ s^−1^. Statistical differences were evaluated using ANOVA *p* ≤ 0.05 followed by Tukey’s HSD post-hoc test. Mean ± SE (*n* = 4).

## References

[1] Neira E. (2002). Chile’s Frontier Forests.

[2] Villagran C., Hinojosa F. (1997). Historia de los bosques del sur de Sudamérica, II: Análisis fitogeográfico. Rev. Chil. Hist. Nat..

[3] Armesto J.J., Rozzi R., Smith-Ramírez C., Arroyo M.T.K. (1998). Conservation Targets in South American Temperate Forests. Science.

[4] Galloway D.J. (1996). Los Líquenes Del Bosque Templado de Chile. Ecología de los Bosques Nativos de Chile.

[5] Woda C., Huber A., Dohrenbusch A. (2006). Vegetación Epifita y Captación de Neblina En Bosques Siempreverdes En La Cordillera Pelada, Sur de Chile. Bosque.

[6] Rodríguez R., Alarcón D., Espejo J. (2009). Helechos Nativos del Centro y sur de Chile.

[7] Marticorena A., Alarcón D., Abello L., Atala C. (2010). Plantas Trepadoras, Epífitas y Parásitas Nativas de Chile.

[8] Ellis C.J. (2012). Lichen Epiphyte Diversity: A Species, Community and Trait-Based Review. Perspect. Plant Ecol. Evol. Syst..

[9] Asplund J., Wardle D.A. (2017). How Lichens Impact on Terrestrial Community and Ecosystem Properties. Biol. Rev..

[10] Sillett S.C., Rambo T.R. (2000). Vertical Distribution of Dominant Epiphytes in Douglas-Fir Forests of Central Oregon Cascades. Northwest Sci..

[11] Li S., Liu W.-Y., Li D.-W., Song L., Shi X.-M., Lu H.-Z. (2015). Species Richness and Vertical Stratification of Epiphytic Lichens in Subtropical Primary and Secondary Forests in Southwest China. Fungal Ecol..

[12] Green T.G.A., Lange O.L., Cowan I.R. (1994). Ecophysiology of Lichen Photosynthesis: The Role of Water Status and Thallus Diffusion Resistances. Cryptogam. Bot..

[13] Sancho L.G., Kappen L. (1989). Photosynthesis and Water Relations and the Role of Anatomy in Umbilicariaceae (Lichenes) from Central Spain. Oecologia.

[14] Lakatos M., Rascher U., Büdel B. (2006). Functional Characteristics of Corticolous Lichens in the Understory of a Tropical Lowland Rain Forest. New Phytol..

[15] Beckett R.P. (1995). Some Aspects of the Water Relations of Lichens from Habitats of Contrasting Water Status Studied Using Thermocouple Psychrometry. Ann. Bot..

[16] Giordani P., Incerti G., Rizzi G., Rellini I., Nimis P.L., Modenesi P. (2014). Functional Traits of Cryptogams in Mediterranean Ecosystems Are Driven by Water, Light and Substrate Interactions. J. Veg. Sci..

[17] Rosabal D., Burgaz A.R., Altamirano A., Aragón G. (2012). Differences in Diversity of Corticolous Lichens between Interior and Edge of the Monte Barranca Semi-Deciduous Forest, Santiago de Cuba. Bryologist.

[18] Paillet Y., Bergès L., Hjältén J., Odor P., Avon C., Bernhardt-Römermann M., Bijlsma R.-J., De Bruyn L., Fuhr M., Grandin U. (2010). Biodiversity Differences between Managed and Unmanaged Forests: Meta-Analysis of Species Richness in Europe. Conserv. Biol..

[19] Dullinger S., Essl F., Rabitsch W., Erb K.-H., Gingrich S., Haberl H., Hülber K., Jarošík V., Krausmann F., Kühn I. (2013). Europe’s Other Debt Crisis Caused by the Long Legacy of Future Extinctions. Proc. Natl. Acad. Sci. USA.

[20] Sancho L.G., de la Torre R., Horneck G., Ascaso C., de Los Rios A., Pintado A., Wierzchos J., Schuster M. (2007). Lichens Survive in Space: Results from the 2005 LICHENS Experiment. Astrobiology.

[21] Raggio J., Pintado A., Ascaso C., De La Torre R., De Los Ríos A., Wierzchos J., Horneck G., Sancho L.G. (2011). Whole Lichen Thalli Survive Exposure to Space Conditions: Results of Lithopanspermia Experiment with Aspicilia Fruticulosa. Astrobiology.

[22] Lange O.L. (1969). Experimentell-Ökologische Untersuchungen an Flechten Der Negev-Wüste. Flora Oder Allg. Bot. Zeitung. Abt. B Morphol. Geobot..

[23] Péli E.R., Lei N., Pócs T., Laufer Z., Porembski S., Tuba Z. (2011). Ecophysiological Responses of Desiccation-Tolerant Cryptobiotic Crusts. Cent. Eur. J. Biol..

[24] Green T.G.A., Sancho L.G., Pintado A., Lüttge U., Beck E., Bartels D. (2011). Ecophysiology of Desiccation/Rehydration Cycles in Mosses and Lichens. Plant Desiccation Tolerance.

[25] Kranner I., Zorn M., Turk B., Wornik S., Beckett R.P., Batič F. (2003). Biochemical Traits of Lichens Differing in Relative Desiccation Tolerance. New Phytol..

[26] Challabathula D., Zhang Q., Bartels D. (2018). Protection of Photosynthesis in Desiccation-Tolerant Resurrection Plants. J. Plant Physiol..

[27] Kershaw K.A., MacFarlane J.D. (1980). Physiological-Environmental Interactions in Lichens. X. Light as an Ecological Factor. New Phytol..

[28] Gauslaa Y., Solhaug K.A. (1996). Differences in the Susceptibility to Light Stress Between Epiphytic Lichens of Ancient and Young Boreal Forest Stands. Funct. Ecol..

[29] Gauslaa Y., Solhaug K.A. (2000). High-Light-Intensity Damage to the Foliose Lichen Lobaria Pulmonaria within Natural Forest: The Applicability of Chlorophyll Fluorescence Methods. Lichenologist.

[30] Beckett R.P., Minibayeva F., Solhaug K.A., Roach T. (2021). Photoprotection in Lichens: Adaptations of Photobionts to High Light. Lichenologist.

[31] Seaward M.R.D., Nash I., Thomas H. (2008). Environmental Role of Lichens. Lichen Biology.

[32] Scheidegger C., Werth S. (2009). Conservation Strategies for Lichens: Insights from Population Biology. Fungal Biol. Rev..

[33] Green T.G.A., Lange O.L., Cowan I.R. (1991). Ecophysiological Adaptations of the Lichen Genera Pseudocyphellaria and Sticta to South Temperate Rainforests. Lichenologist.

[34] Lange O.L., Kilian E., Ziegler H. (1986). Water Vapor Uptake and Photosynthesis of Lichens: Performance Differences in Species with Green and Blue-Green Algae as Phycobionts. Oecologia.

[35] Araya-Osses D., Casanueva A., Román-Figueroa C., Uribe J.M., Paneque M. (2020). Climate Change Projections of Temperature and Precipitation in Chile Based on Statistical Downscaling. Clim. Dyn..

[36] Atala C., Schneider C., Bravo G., Quilodrán M., Vargas R. (2015). Anatomical, Physiological and Chemical Differences between Populations of *Pseudocyphellaria flavicans* (Hook. f. & Taylor) Vain. from Chile. Gayana Bot..

[37] González-Reyes Á., Muñoz A.A. (2013). Cambios En La Precipitación de La Ciudad de Valdivia (Chile) Durante Los Últimos 150 Años. Bosque.

[38] Sillett S.C., McCune B. (1998). Survival and Growth of Cyanolichen Transplants in Douglas-Fir Forest Canopies. Bryologist.

[39] Villagra J., Montenegro D., San Martín C., Ramírez C., Álvarez I. (2009). Estudio de La Flora Liquénica de Las Turberas de La Comuna de Tortel (Región de Aisén), Patagonia Chilena. An. Inst. Patagon..

[40] Bustamante R., Serey I., Guzmán G. (1989). Distribución y Abundancia de Epífitos En Bosques de Lenga (Nothofagus Pumilio), Isla Navarino, Región de Magallanes y de La Antártica Chilena. Ser. Cient. Inst. Antárt. Chil..

[41] Rubio C., Saavedra M., Cuéllar M., Díaz R., Quilhot W. (2013). Epiphytic Lichens of Conguill{io National Park, Soutern Chile. Gayana Bot..

[42] McCune B. (2000). Lichen Communities as Indicators of Forest Health. Bryologist.

[43] Hauck M., Meißner T. (2002). Epiphytic Lichen Abundance on Branches and Trunks of Abies Balsamea on Whiteface Mountain, New York. Lichenologist.

[44] Marmor L., Tõrra T., Saag L., Randlane T. (2012). Species Richness of Epiphytic Lichens in Coniferous Forests: The Effect of Canopy Openness. Ann. Bot. Fenn..

[45] Díaz I.A., Sieving K.E., Peña-Foxon M.E., Larraín J., Armesto J.J. (2010). Epiphyte Diversity and Biomass Loads of Canopy Emergent Trees in Chilean Temperate Rain Forests: A Neglected Functional Component. For. Ecol. Manag..

[46] Normann F., Weigelt P., Gehrig-Downie C., Gradstein S.R., Sipman H.J.M., Obregon A., Bendix J. (2010). Diversity and Vertical Distribution of Epiphytic Macrolichens in Lowland Rain Forest and Lowland Cloud Forest of French Guiana. Ecol. Indic..

[47] Barkman J.J. (1969). Phytosociology and Ecology of Cryptogamic Epiphytes (Including a Taxonomic Survey and Description of Their Vegetation Units in Europe).

[48] Rambo T.R. (2010). Structure and Composition of Corticolous Epiphyte Communities in a Sierra Nevada Old-Growth Mixed-Conifer Forest. Bryologist.

[49] Villagra J., Sancho L.G., Alors D. (2023). Macrolichen Communities Depend on Phorophyte in Conguillío National Park, Chile. Plants.

[50] Parra M.J., Acuña K., Corcuera L.J., Saldaña A. (2009). Vertical Distribution of Hymenophyllaceae Species among Host Tree Microhabitats in a Temperate Rain Forest in Southern Chile. J. Veg. Sci..

[51] Gauslaa Y., Ohlson M., Solhaug K.A., Bilger W., Nybakken L. (2001). Aspect-Dependent High-Irradiance Damage in Two Transplanted Foliose Forest Lichens, Lobaria Pulmonaria and Parmelia Sulcata. Can. J. For. Res..

[52] Green T.G.A., Nash T.H., Lange O.L., Nash I., Thomas H. (2008). Physiological Ecology of Carbon Dioxide Exchange. Lichen Biology.

[53] Kranner I., Beckett R., Hochman A., Nash T.H. (2008). Desiccation-Tolerance in Lichens: A Review. Bryologist.

[54] Ndhlovu N.T., Minibayeva F., Smith F.R., Beckett R.P. (2023). Lichen Substances Are More Important for Photoprotection in Sun than Shade Collections of Lichens from the Same Species. Bryologist.

[55] Galloway D.J. (1992). Studies in Pseudocyphellaria (Lichens) III. The South American Species. Bibl. Lichenol..

[56] Kermit T., Gauslaa Y. (2001). The Vertical Gradient of Bark pH of Twigs and Macrolichens in a Picea Abies Canopy Not Affected by Acid Rain. Lichenologist.

[57] Schneider P.H., Schmitt J.L. (2011). Composition, Community Structure and Vertical Distribution of Epiphytic Ferns on Alsophila Setosa Kaulf., in a Semideciduous Seasonal Forest, Morro Reuter, RS, Brazil. Acta Bot. Bras..

[58] Demmig-Adams B., Máguas C., Adams W.W., Meyer A., Kilian E., Lange O.L. (1990). Effect of High Light on the Efficiency of Photochemical Energy Conversion in a Variety of Lichen Species with Green and Blue-Green Phycobionts. Planta.

[59] Lüttge U. (2013). Desiccation Tolerance of the Epilithic Lichen Lecanora Muralis (Schreb.) Rabenh. in the Temperate Climate. Flora—Morphol. Distrib. Funct. Ecol. Plants.

[60] Garreaud R.D. (2011). Cambio Climático: Bases Físicas e Impactos en Chile.

[61] Martínez-Retureta R., Aguayo M., Abreu N.J., Stehr A., Duran-Llacer I., Rodríguez-López L., Sauvage S., Sánchez-Pérez J.-M. (2021). Estimation of the Climate Change Impact on the Hydrological Balance in Basins of South-Central Chile. Water.

[62] Luebert F., Pliscoff P. (2006). Sinopsis Bioclimática y Vegetacional de Chile—Facultad de Ciencias Forestales y de la Conservación de la Naturaleza.

[63] Di Castri F., Hajek E.R. (1976). Representaciones gráficas de pares climáticas. Bioclimatología de Chile.

[64] Gatica A., Pereira Í., Vallejos O. (2011). Líquenes epífitos: Una herramienta para estudiar la continuidad ecológica en Isla Mocha, Chile. Gayana Bot..

[65] Green T.G.A., Büdel B., Meyer A., Zellner H., Lange O.L. (1997). Temperate Rainforest Lichens in New Zealand: Light Response of Photosynthesis. N. Z. J. Bot..

[66] Pardow A., Hartard B., Lakatos M. (2010). Morphological, Photosynthetic and Water Relations Traits Underpin the Contrasting Success of Two Tropical Lichen Groups at the Interior and Edge of Forest Fragments. AoB Plants.

[67] Pintado A., Sancho L.G., Green T.G.A., Blanquer J.M., Lázaro R. (2005). Functional Ecology of the Biological Soil Crust in Semiarid SE Spain: Sun and Shade Populations of Diploschistes Diacapsis (Ach.) Lumbsch. Lichenologist.

[68] Reiter R., Höftberger M., Allan Green T.G., Türk R. (2008). Photosynthesis of Lichens from Lichen-Dominated Communities in the Alpine/Nival Belt of the Alps—II: Laboratory and Field Measurements of CO2 Exchange and Water Relations. Flora Morphol. Distrib. Funct. Ecol. Plants.

[69] Gauslaa Y., Palmqvist K., Solhaug K.A., Holien H., Hilmo O., Nybakken L., Myhre L.C., Ohlson M. (2007). Growth of Epiphytic Old Forest Lichens across Climatic and Successional Gradients. Can. J. For. Res..

[70] Gauslaa Y., Goward T. (2012). Relative Growth Rates of Two Epiphytic Lichens, Lobaria Pulmonaria and Hypogymnia Occidentalis, Transplanted within and Outside of Populus Dripzones. Botany.

[71] Heber U., Bilger W., Bligny R., Lange O.L. (2000). Phototolerance of Lichens, Mosses and Higher Plants in an Alpine Environment: Analysis of Photoreactions. Planta.

[72] Veblen T.T., Donoso C., Kitzberger T., Robertus A.J. (1996). Ecology of Southern Chilean and Argentinean Nothofagus Forests. The Ecology and Biogeography of Nothofagus Forests.

[73] Oyarzun C.E., Godoy R., Sepulveda A. (1998). Water and Nutrient Fluxes in a Cool Temperate Rainforest at the Cordillera de La Costa in Southern Chile. Hydrol. Process..

[74] Galloway D.J. (1994). Studies on the Lichen Genus Sticta (Schreber) Ach.: I. Southern South American Species. Lichenologist.

[75] Wedin M. (1995). The Lichen Family Sphaerophoracae (Caliciales, Ascomycotina) in Temperate Areas of the Southern Hemisphere.

[76] Parmasto E. (1978). Nova Hedwigia.

[77] Rascher U., Liebig M., Lüttge U. (2000). Evaluation of Instant Light-Response Curves of Chlorophyll Fluorescence Parameters Obtained with a Portable Chlorophyll Fluorometer on Site in the Field. Plant Cell Environ..

[78] Barnes J.D., Balaguer L., Manrique E., Elvira S., Davison A.W. (1992). A Reappraisal of the Use of DMSO for the Extraction and Determination of Chlorophylls a and b in Lichens and Higher Plants. Environ. Exp. Bot..

[79] Ralph P.J., Gademann R. (2005). Rapid Light Curves: A Powerful Tool to Assess Photosynthetic Activity. Aquat. Bot..

[80] Maxwell K., Johnson G.N. (2000). Chlorophyll Fluorescence—A Practical Guide. J. Exp. Bot..

[81] Jensen M., Kranner I.C., Beckett R.P., Varma A.K. (2002). Measurement of Chlorophyll Fluorescence in Lichens. Protocols in Lichenology: Culturing, Biochemistry, Ecophysiology and Use in Biomonitoring.

[82] Sokal R.R., Rohlf F.J. (1970). Biometry: The Principles and Practice of Statistics in Biological Research.

