# Supplementary material for: Desiccation Tolerance of Epiphytic Macrolichens in an Evergreen Temperate Rain Forest (Alerce Costero National Park, Chile)
Source: Plants (Basel). 2024 May 31;13(11):1519. doi: 10.3390/plants13111519 (PMC11174617; doi:10.3390/plants13111519)
Supplement: Supplementary file 1 [file plants-13-01519-s001.zip › plants-2986951-supplementary.pdf]

Supplementary Table S1. Concentration of total chlorophyll (Chl) and carotenes (car) in epiphytic macrolichens in the time (1, 13, 24, 53, 71 days of experiment). The pigment concentration is referred as mg/g. Mean ± SE (n=4). ANOVA with repeated measures was used to compare concentration pigment over time and different letters indicate significant differences. ANOVA p≤ 0.05, followed by Tukey's HSD post-hoc test. Mean ± SE (n=12).

Time (days)	Species	Chl mg/g	Car mg/g	Species	Chl mg/g	Car mg/g
P		0.036	0.009		0.003	0.019
1		0.84 ± 0.13 a	1.05 ± 0.16 a		1.17 ± 0.27 a	1.09 ± 0.23 a
13	*P. coerulescens*	0.70 ± 0.02 ab	0.92 ± 0.02 ab	*L. tenerum*	0.88 ± 0.05 ab	0.82 ± 0.05 ab
24		0.63 ± 0.12 ab	0.86 ± 0.07 ab		0.64 ± 0.23 abc	0.59 ± 0.20 abc
53		0.49 ± 0.13 ab	0.60 ± 0.16 ab		0.30 ± 0.03 b	0.30 ± 0.02 b
71		0.37 ± 0.04 b	0.50 ± 0.04 b		0.16 ± 0.03 c	0.16 ± 0.02 c
P		0.225	0.225		0.044	0.032
1		1.05 ± 0.03	1.13 ± 0.04		0.82 ± 0.15 a	0.78 ± 0.12 a
13	*S. caulescens*	0.60 ± 0.20	0.66 ± 0.14	*B. australe*	0.37 ± 0.09 ab	0.39 ± 0.10 ab
24		1.12 ± 0.19	1.17 ± 0.21		0.68 ± 0.25 ab	0.62 ± 0.22 ab
53		1.04 ± 0.10	1.03 ± 0.15		0.33 ± 0.09 ab	0.32 ± 0.09 ab
71		1.02 ± 0.15	1.24 ± 0.13		0.23 ± 0.05 b	0.17 ± 0.03 b
P		<0.001	<0.001		0.12	0.11
1		1.94 ± 0.26 ab	2.47 ± 0.15 a		1.46 ± 0.23	1.35 ± 0.20
13	*P. berberina*	2.15 ± 0.13 a	2.62 ± 0.04 a	*P. divulsa*	1.25 ± 0.23	1.23 ± 0.20
24		1.28 ± 0.14 bc	1.85 ± 0.10 b		1.69 ± 0.23	1.64 ± 0.22
53		0.71 ± 0.14 c	1.35 ± 0.14 bc		1.53 ± 0.15	1.41 ± 0.12
71		0.60 ± 0.04 c	1.19 ± 0.12 c		1.03 ± 0.13	1.02 ± 0.11
P		0.683	0.529		0.04	0.026
1		1.01 ± 0.06	0.93 ± 0.05		2.22 ± 0.12 a	1.93 ± 0.10 a
13	*P. nitida*	1.07 ± 0.20	0.90 ± 0.18	*S. ainoae*	1.86 ± 0.10 ab	1.71 ± 0.08 ab
24		1.00 ± 0.16	0.88 ± 0.12		1.85 ± 0.16 ab	1.66 ± 0.14 ab
53		0.78 ± 0.24	0.63 ± 0.19		1.54 ± 0.08 ab	1.43 ± 0.05 ab
71		0.79 ± 0.13	0.69 ± 0.12		1.44 ± 0.22 b	1.24 ± 0.19 b

Supplementary Table S2. Maximum electron transport rate (ETRmax) and saturation points (PPDsat) obtained from the exponential fit (using the Sigma Plot Program) of the data recorded in situ. The PPFD units are referred to as μmol m^{-2} s^{-1}. Statistical differences were evaluated using ANOVA $p \leq 0.05$ followed by Tukey's HSD post-hoc test. Mean ± SE (n=4).

Species	ETRmax p= 0.016	PPFDsat p= 0.023	R^2
P. coerulescens	5.28 ± 0.91 ab	31.18 ± 4.11 a	0,78 ± 0,12
S. caulescens	7.21 ± 1.80 ab	53.76 ± 10.23 ab	0,74 ± 0,07
L. tenerum	5.66 ± 0.69 ab	24.73 ± 8.51 a	0,78 ± 0,01
B. australe	6.36 ± 1.19 ab	36.56 ± 4.10 ab	0,87 ± 0,78
P. berberina	5.48 ± 0.72 ab	38.35 ± 6.83 ab	0,95 ± 0,02
P. nitida	10.42 ± 1.74 b	87.09 ± 23.2 b	0,88 ± 0,03
P. divulsa	4.06 ± 0.52 a	32.61 ± 8.75 a	0,74 ± 0,07
S. ainoae	4.59 ± 0.49 a	37.99 ± 3.37 ab	0,88 ± 0,05
